# Genotype Reconstruction of Paternity in European Lobsters (*Homarus gammarus*)

**DOI:** 10.1371/journal.pone.0139585

**Published:** 2015-11-13

**Authors:** Charlie D. Ellis, David J. Hodgson, Carl André, Tonje K. Sørdalen, Halvor Knutsen, Amber G. F. Griffiths

**Affiliations:** 1 Environment and Sustainability Institute, University of Exeter, Penryn Campus, Penryn, Cornwall, TR10 9FE, United Kingdom; 2 National Lobster Hatchery, South Quay, Padstow, Cornwall, PL28 8BL, United Kingdom; 3 Centre for Ecology and Conservation, University of Exeter, Penryn Campus, Penryn, Cornwall, TR10 9FE, United Kingdom; 4 Department of Marine Ecology—Tjärnö, University of Gothenburg, 452–96, Strömstad, Sweden; 5 University of Agder, N-4604, Kristiansand, Norway; 6 Institute of Marine Research, Flødevigen, 4817, His, Norway; 7 Centre for Ecological and Evolutionary Synthesis, Department of Biosciences, University of Oslo, P.O. Box 1066, Blindern, N-0316, Oslo, Norway; Macquarie University, AUSTRALIA

## Abstract

Decapod crustaceans exhibit considerable variation in fertilisation strategies, ranging from pervasive single paternity to the near-ubiquitous presence of multiple paternity, and such knowledge of mating systems and behaviour are required for the informed management of commercially-exploited marine fisheries. We used genetic markers to assess the paternity of individual broods in the European lobster, *Homarus gammarus*, a species for which paternity structure is unknown. Using 13 multiplexed microsatellite loci, three of which are newly described in this study, we genotyped 10 eggs from each of 34 females collected from an Atlantic peninsula in the south-western United Kingdom. Single reconstructed paternal genotypes explained all observed progeny genotypes in each of the 34 egg clutches, and each clutch was fertilised by a different male. Simulations indicated that the probability of detecting multiple paternity was in excess of 95% if secondary sires account for at least a quarter of the brood, and in excess of 99% where additional sire success was approximately equal. Our results show that multiple paternal fertilisations are either absent, unusual, or highly skewed in favour of a single male among *H*. *gammarus* in this area. Potential mechanisms upholding single paternal fertilisation are discussed, along with the prospective utility of parentage assignments in evaluations of hatchery stocking and other fishery conservation approaches in light of this finding.

## Introduction

The reproductive behaviour and ecology of fished species can affect their vulnerability to population collapses, and their subsequent ability to recover [1]. Polyandry may arise in breeding females as a life history strategy in order to increase the genetic diversity or fitness of offspring [2,3], or where males are sperm limited [4]. Selective fishing may also influence the occurrence of polyandry, especially where mating strategies are dependent on age, size, or sex ratio [1,5,6]. As a result, information on the dynamics of female mating strategies is a vital component to the informed conservation management of exploited fisheries [7].

Clutch fertilisation in marine decapods varies between species and populations, from pervasive single paternity (e.g. snow crab [8]) to ubiquitous multiple paternity (e.g. squat lobsters [9]). Multiple sires have been detected within individual clutches in a variety of aquatic crustaceans (e.g. ghost shrimp [10]; Norway lobster [11]; porcelain crab [12]; Dungeness crab [13]; rock shrimp [14]; freshwater crayfishes [15]; Pacific gooseneck barnacle [16]). However, the frequency of polyandrous fertilisation remains unknown in the European lobster (*Homarus gammarus*), a high-value species exploited extensively throughout its range by trap fishing. The presence of multiple paternal fertilisations has been detected among individual egg clutches of the closely-related American lobster, *Homarus americanus* [17,18], with some evidence from the wild that increased fishing pressure disrupts the natural monandrous behaviour of some females via reductions in the abundance, size or post-copulatory mate-guarding ability of breeding males [18].

Despite supporting a highly lucrative fishery, information on the reproductive ecology of *H*. *gammarus* in the wild is scarce [19], and is often implied from that of the better-studied *H*. *americanus*. Female *H*. *americanus* are thought to seek out and compete for males and usually moult during a period of shelter cohabitation, whereupon a spermatophore is deposited by the male into the seminal receptacle of the female [20,21]. The male attempts to prevent further insemination from competitors by guarding the female until both her shell and a sperm plug blocking the entrance to the seminal receptacle have hardened [20,22]. Females vacate the male’s shelter and usually store the spermatophore for approximately a year before spawning, whereupon it is released to externally fertilise the eggs during extrusion and oviposition [23,24]. Homarid eggs hatch following 9–11 months of development while stored ventrally along the female abdomen, at which point most mature females mate and moult again, forming a biennial reproductive cycle [21,24]. Occasionally females moult, mate and spawn annually [24], while large (>120 mm carapace length [CL]) females can go several years without moulting and may mate during intermoult if spermatophore reserves are insufficient to sire a brood [25].

It has long been established that female fecundity increases with increasing body size [24,26,27], and studies on the effects of male size in other lobster species show that ejaculate load is also size-specific and may be reduced by previous copulations [20,28]. Where the abundance and mean size of males is reduced by fishing, it has been proposed that the population may become sperm limited, with the production of larvae restricted by a lack of available spermatophore with which to fertilise the maximum egg capability of breeding females [28]. Such sperm limitation may cause females to seek additional copulations, with more than one spermatophore used to fertilise an egg clutch [18,20]. Alongside sperm limitation, other hypotheses proposed to explain observed multiple paternity in marine invertebrates have included convenience polyandry [29–31] and enforced mating [14]. Where multiple paternity has been identified among marine crustaceans, considerable skews in fertilisation success towards a single male have often been detected [9,14,16,18]. This has been proposed to result from various post-copulatory processes including spermatophore stratification [32], cryptic female choice [30] and sperm competition, although the latter was ruled out for *H*. *americanus* because their sperm lack motility [18,22].

We investigated *H*. *gammarus* paternity around Cornwall, an Atlantic peninsula in south-western UK, where lobsters are intensively fished and are also the focus of stock enhancement by a local hatchery [33]. Because physical tags having proven largely ineffective in marking early-stage post-larval lobsters [34–36], the hatchery is interested in pursuing genetic methods of parentage assignment that have allowed the successful identification of stocked finfish among admixed wild populations [37–39]. The tissue archiving requirements and general suitability of such an application are in part dependent on the number of sires contributing to individual clutches, adding to the need for information of lobster paternity in the region. By reconstructing male genotypes from clutches of fertilised eggs, we aimed to estimate the frequency of multiple paternity and thus elucidate the typical fertilisation scenario in lobsters from this important regional fishery.

## Materials and Methods

### Ethics statement

Permission to obtain tissue samples from adult lobsters (for both paternity assays and population screening) were obtained from the Cornwall Inshore Fisheries Conservation Authority (IFCA), who regulate and manage the lobster fishery within coastal waters. Tissue samples were collected on board commercial vessels as part of regular fishing routines. The collection of tissue samples from adult lobsters from the Isles of Scilly did not require the permission of the Isles of Scilly IFCA since samples were obtained from animals already landed to a merchant on the mainland. Eggs for paternity assays were collected from ovigerous females captured within the six nautical mile inshore jurisdiction of Cornwall IFCA, who provided written permission for both the sampling of eggs and the temporary landing of ovigerous lobsters, which is normally prohibited by a regional bylaw [40]. The European lobster is categorised as being of Least Concern in the Red List of Threatened Species of the International Union for Conservation of Nature [41].

### Sites and sampling

**Fig 1:**
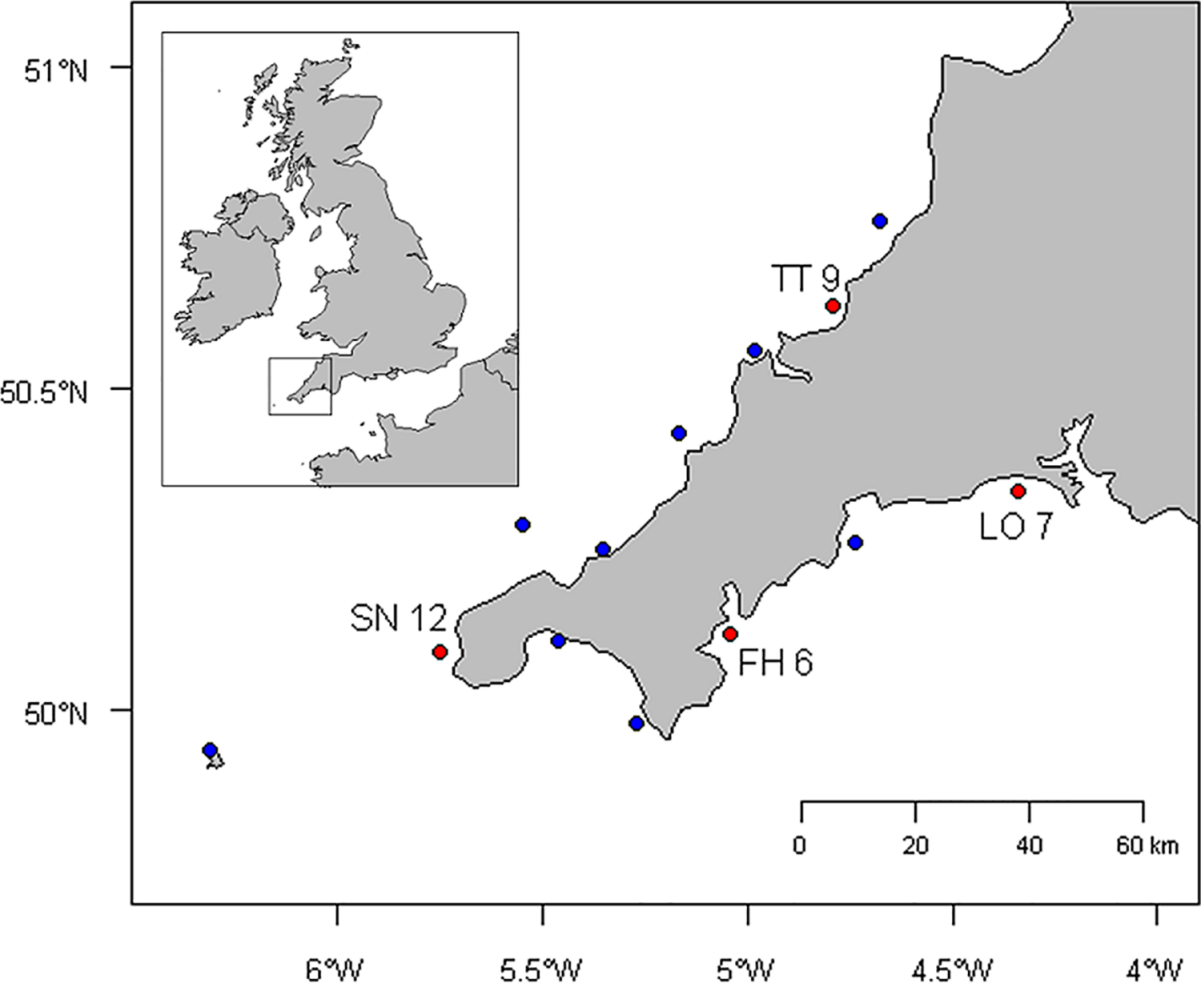
Map of sample sites. Map of the Cornwall peninsula showing the location of sampling sites. Red points denote the paternity sample sites Tintagel (TT), Sennen (SN), Falmouth (FH) and Looe (LO), with sample sizes denoting the number of clutches successfully tested. These four sites, and nine additional sites denoted by blue points, were each used to sample 24 individuals to provide accurate estimates of regional allele frequencies. Position relative to the UK, Ireland and continental Europe is inset.

During March and April 2013, trap-caught ovigerous female lobsters were collected directly from selected inshore fishers temporarily permitted to land these animals by the regional fisheries management authority. Typically, the rocky Celtic Sea habitats to the north and far west support a greater abundance of lobster than the mixed substrates of the western English Channel along the southern coast [42]. As such, lobsters were sourced from two sites in each area (four sites in total, separated by a minimum Euclidean distance of 55 km) to account for any spatial variation in paternity structure (Fig 1). Where possible, samples were taken immediately upon receipt of the lobsters, although occasionally they were stored in holding tanks for a maximum of 48 hours before sampling. Sampling consisted of the removal of a small piece of maternal tissue from the tip of a hindmost pleopod, and of ten eggs from the clutch (total clutch size is specific of female size and even region, though is typically 9–13,000 for mean-sized individuals of 103 mm CL [27]). An egg was removed from both the base and the tip of the egg-mass from each of the five pairs of pleopods. Egg sampling was structured in this way to maximise the likelihood of detecting multiple paternity and because some marine decapods (though not *H*. *americanus* [18]) have demonstrated spatial segregation of multiple paternal fertilisations [9,10]. Twelve females were sampled from each of two Celtic Sea and English Channel locations, although insufficient DNA yields from undeveloped eggs later reduced these sample sizes. As such, 340 eggs from 34 females were genotyped successfully (Fig 1). Female carapace length (CL) was measured using a Vernier caliper and rounded down to the nearest whole millimetre, as per [43]. The assessment of a wide range of female sizes is important given the expectation that the frequency of multiple paternity may vary with female size, particularly if caused by sperm-limitation [20,28].

### Microsatellite genotyping

Genotyping of tissue samples was carried out using 15 microsatellite loci; 12 previously published [19], and the three newly characterised loci (see S1 Text for development process). Maternal DNA was extracted from individual pleopod tissues and progeny DNA from whole eggs using the Wizard^®^ SV 96 Genomic DNA Purification System (Promega). Primer oligonucleotides were synthesized by Eurofins Genomics (Eurofins Genomics), with forward primers 5’-tagged with one of four fluorescent sequencing dyes; FAM, ATTO 550, ATTO 565 and Yakima Yellow. The Mulitplex PCR Kit (Qiagen) was used to allow the amplification of all loci across four multiplexes (See Table 1 for multiplex organisation). PCR volumes of 8 μl were prepared in the following reaction mix: 4 μl Multiplex PCR Mix; forward and reverse primers at 0.48–1.33 μM (Multiplex 1, 0.88 μM, apart from HGD106, 0.48 μM; Multiplex 2, 1.00 μM; Multiplexes 3 and 4, 1.33 μM); and 2 μl template DNA (20–50 ng). PCR was conducted in a Techne Prime Elite 96 thermocycler (Bibby Scientific Ltd.), with an initial denaturation (94°C, 3 min), then 35 cycles of denaturation (94°C, 40 s), annealing (55°C, 40 s) and extension (72°C, 30 s), before a final extension (72°C, 4 min). Fragment analysis was carried out for the 312 samples using an ABI 3130 Genetic Analyzer (Applied Biosystems Inc.). Alleles were automatically sized against Genescan™ 500 LIZ™ size standard (Applied Biosystems Inc.) using Geneious 6.1 software (Biomatters Ltd.), before also being checked manually and rescored where necessary.

**Table 1:** Loci exclusion probabilities.

Rank	Locus	Multiplex	Exclusion Probability	
			Maternal genotype known	Neither parental genotype known
1	HGC120	4	0.732	0.575
2	HGC131b	4	0.662	0.491
3	HGD110	4	0.611	0.435
4	HGC111	3	0.494	0.314
5	HGB6	2	0.483	0.308
6	HGD106	1	0.481	0.301
7	HGC103	2	0.476	0.304
8	HGB4	1	0.430	0.251
9	HGC118	1	0.378	0.201
10	HGD111	3	0.350	0.186
11	HGD129	2	0.347	0.179
12	HGD117	1	0.320	0.178
13	HGC6	2	0.212	0.071
14	HGA8 ^a^	1	*0*.*647*	*0*.*473*
15	HGC129 ^a^	3	*0*.*543*	*0*.*363*

Loci are ranked via individual exclusion probabilities, assuming an assay of 10 progeny genotypes and deriving allele frequencies from a regional population survey (see S1 Text for sampling details).

^a^Loci which were removed from paternity analyses due to the presence of null alleles; as such these are ranked last and their exclusion probabilities (italicised) will be inaccurate.

While some studies have previously pooled eggs from each pleopod region or the whole clutch into single extractions, we elected to genotype eggs individually. Pooling progeny genotypes can allow the detection of multiple paternity while boosting the number of progeny screened and the sample size of females per unit effort, but such an approach can significantly underestimate the true number of sires [9] and provides no way of estimating fertilisation skew. To prevent genotyping errors overestimating the occurrence of multiple paternity, any progeny genotype that did not support a single paternal contribution (i.e. where three or more alleles were recorded at a locus) was retested in single-locus PCR (using Qiagen Taq PCR Master Mix in place of Multiplex PCR Mix) and controlled fragment analysis procedures. The software FreeNA [44] was used to estimate the frequency of null alleles from regional population genotype data of 312 individuals (see S1 Text for sampling details).

### Statistical analysis

Probabilities of detecting multiple paternal contributions (PrDM) were quantified by the software *PrDM* [45]. Using regional population allele frequencies (from 312 individuals–see S1 Text for sampling details), *PrDM* used Monte Carlo simulations to calculate PrDM under various scenarios of skew between the fertilisation contributions of multiple males; two males in ratios of 50:50, 60:40, 70:30, 80:20 and 90:10, and three males in ratios of 34:33:33, 50:25:25, 60:20:20, 70:15:15, 80:10:10 and 90:5:5. The software GERUD 2.0 [46] was used to estimate the exclusion probabilities (the probability that they exclude an unrelated individual from a putative pedigree [47]) of individual loci to enable loci to be ranked by power to assign parentage. GERUD 2.0 was used to reconstruct the minimum number of possible paternal genotypes, which were also independently assembled manually from progeny genotypes. Because GERUD 2.0 only reconstructs the minimum number of unknown parental contributions that can explain the progeny genotypes, two-allele genotypes are presumed to be heterozygotes. Although unlikely given the number of markers used, it is therefore possible that two males displaying only homozygote or shared alleles would be reconstructed as a single male. As such, total heterozygosity calculations and heterozygote excess tests were carried out on pooled parental genotypes using GENEPOP 4.2 software [48]. The presence of heterozygote excess or significantly increased heterozygosity compared to known maternal genotypes could suggest an underestimation of the number of males contributing to reconstructed paternal genotypes.

## Results

### Egg DNA yields and female sizes

All eggs in intermediate and later stages of development (as evidenced by brown and red colouration) yielded suitable quantities of DNA for downstream analysis. However, 3 of 24 Celtic Sea females and 11 of 24 English Channel females possessed eggs that were either unfertilised [49] or in early stages of development (as evidenced by black and/or dark green colouration) from which DNA yields were insufficient to allow successful genotyping, reducing the actual sample sizes to 21 and 13 respectively. Of those females providing successful progeny arrays, size (CL) ranged from 94–155 mm (*n*_Total_ = 34, mean CL = 113.5 mm, SE ±2.31), with English Channel individuals (mean CL = 117.9 mm, SE ±4.26) tending to be slightly larger than those from Celtic Sea sites (mean = 110.7 mm, SE ±2.56).

### Genotyping and marker power

Maternal and progeny samples that amplified effectively were screened at all 15 loci, however two loci were dropped from the analysis upon the detection of null alleles, which are known to introduce substantial errors in empirical assessments of parentage [50–52]. In this case, null alleles appear to have caused mismatches between maternal and progeny genotypes, or progeny genotypes to suggest three paternal alleles at the loci HGA8 and HGC129 (in 11 and four occasions among 68 parents, respectively). FreeNA confirmed null alleles at frequencies of 0.11 for HGA8 and 0.04 for HGC129. Null allele frequencies were zero for all other loci except HGC103 and HGD111, for which negligible frequencies of 0.02 were estimated. Because of this, only the remaining 13 markers were used in the determination of potential paternal genotypes and PrDM. The exclusion probabilities of these individual loci ranged from 0.21 to 0.73 when using ten progeny arrays and a known maternal genotype (Table 1). Note that this probability is not a measurement of the likelihood of individual loci successfully detecting multiple paternity or determining the number of sires, but of their likelihood to correctly exclude unrelated males from potential parentage via genotypic mismatch (e.g. when surveying paternal candidates). As such it is indicative of the relative power provided by each locus. The three most powerful loci were HGC120, HGC131b and HGD110.

### Probability of detecting multiple paternity

**Fig 2:**
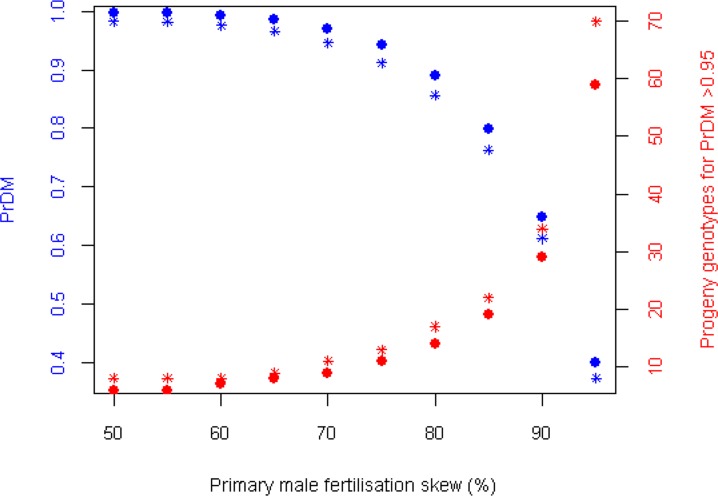
PrDM with skewed male fertilisation success. Variation in PrDM from 10 progeny genotypes (blue axis and data points) and the number of progeny genotypes required to achieve a 95% confidence level in PrDM (red axis and data points) under various scenarios of male fertilisation skew. Round points infer progeny genotyping at all 13 loci, while starred points infer progeny genotyping at only the three most informative loci (all amplified within Multiplex 4).

With 10 progeny genotyped at 13 loci, the probability of detecting a secondary paternal contribution where one was present exceeded 0.99 assuming equal fertilisation contributions (Fig 2). The confidence threshold for the detection of additional males dropped below 95% only when the paternal contribution of secondary sires accounted for 25% or fewer of the progeny. If the paternal contribution had been highly skewed in favour of a primary male in this way, then more than 10 progeny genotypes would have been required to retain a 95% confidence level in PrDM (Fig 2). In scenarios where secondary contributions were split between two males (three sires in total), PrDM effectively remained unchanged, although for some scenarios, one or two fewer progeny genotypes could still yield PrDM >0.95 (S5 Table). Estimates of PrDM based on genotyping at only the three most polymorphic loci (all amplified within Multiplex 4) were almost as powerful as those attained by all 13 loci. PrDM was <0.95 at a lower paternal skew (70:30 as opposed to 75:25), but was only decreased by 0.002–0.037 under the fertilisation scenarios investigated.

### Paternal reconstruction

Reconstructions of paternal genotypes by GERUD 2.0 showed that single male genotypes explained all of the 34 progeny arrays. Of the candidate paternal genotypes, 28 were able to be reconstructed in full at all 13 loci (S4 Table). For six reconstructed paternal genotypes, it was not possible for GERUD 2.0 to resolve the paternal genotype at all 13 loci; four reconstructions were unable to determine paternal genotype at one locus and two more were unresolved at two loci. In these instances, both maternal and paternal genotypes were heterozygous and the paternal genotype possessed one allele that was shared with a maternal allele, but the progeny array contained no homozygotes to determine which allele was shared. On such occasions, GERUD 2.0 simply returned multiple single-sire genotypes that could explain the progeny array which were ranked in order of likelihood according to Mendelian segregation probability. All reconstructed male genotypes differed at multiple loci; no paternal genotype matched those provided by any other progeny array, so the clutches of all 34 females appeared to have been fertilised by 34 separate males. Total heterozygosity of reconstructed paternal genotypes was 0.68, while known maternal genotypes had a total heterozygosity of 0.69. A test for heterozygous excess among reconstructed paternal genotypes was non-significant (p = 0.50) and comparable to that obtained for known maternal genotypes (p = 0.49). Twelve allele scores (1.6%) were altered after genotyping was repeated. Had the original scores been analysed, it would have led to four incidences of multiple paternity (all with 1/10 progeny supporting a second sire).

## Discussion

Unlike many other genetic studies on aquatic crustaceans [9–16,18], our investigation found no evidence for multiple paternal fertilisations of individual *H*. *gammarus* broods. The loci employed ensured the statistical power to detect additional paternal fertilisations was consistently high, exceeding 99% when assuming approximately equal male representation among the progeny, and exceeding 95% wherever secondary males accounted for at least a quarter of the brood. This power to detect secondary sires is greater than that reported by Bailie *et al* [9], which failed to reach 95% at any fertilisation skew when genotyping up to 86 galatheid squat lobster progeny at only two or three microsatellites, and is commensurate with that of Gosselin *et al* [18] for *H*. *americanus* at equal (50:50) skews, but not at extreme (90:10) skews due to our genotyping fewer eggs. The power to detect secondary paternal genotypes with low progeny representation is important since multiply-sired crustacean broods often show high levels of paternal skew, with Bailie *et al* [9] estimating that secondary paternal fertilisations composed 14% or fewer of the majority of galatheid broods. Due to the statistical power of our method falling outside of 95% confidence limits at high paternal fertilisation skews, it is possible that multiple paternity was present but undetected in *H*. *gammarus* broods we assessed. It is unlikely, however; most (64%) multiply-sired broods identified by Gosselin *et al* [18] exhibited secondary fertilisation contributions at ratios where detection probability would have exceeded 95% in our study. Even applying the least frequent rate of detection in a sub-population (11%) and the maximum skew (90:10) found among multiply-sired *H*. *americanus* clutches [18], we would still anticipate at least three cases of multiple paternity among our *H*. *gammarus* samples (two from Celtic Sea sites and one from English Channel sites), of which our power of detection (65%) would have been expected to overlook only one. Overall, our results suggest that multiple paternity is likely to be absent, or rare and highly skewed in favour of a dominant male, among *H*. *gammarus* in this geographical region.

While the reconstruction of paternal genotypes was conservative in that it provides the minimum number of males required to explain the observed progeny genotypes, it appears to be have been accurate in confirming single paternity. Overall heterozygosity of reconstructed paternal genotypes was equal to that of all maternal individuals, and showed no evidence of heterozygous excess, suggesting no underestimation of the number of sires represented among paternal reconstructions. Alongside reconstructing sire contributions from individual egg genotypes, some studies have inferred multiple paternity via significant departures of progeny genotypes from Mendelian expectations of allele frequencies [9]. However, this method was not considered for our analysis because it was deemed potentially ambiguous and unlikely to prove informative given the size of the progeny array per brood, and because the possibility of missing additional paternal alleles across 13 loci was remote.

The prevalence of single paternity among individual *H*. *gammarus* broods suggests that either (i) all females copulated only with a single male; or (ii) females copulated with more than one male, but fertilisation was attained by only a single male.

In *H*. *americanus*, regular monandrous mating appears to be maintained by both female choice (female preference for the protection and/or spermatophore of dominant males [53]) and male competition (male efforts to prevent rival inseminations prior to the formation of a sperm plug [18]). Clear evidence of female choice has also been observed in *H*. *gammarus* [54], so the same processes may well occur in both species. Where polyandry was found in *H*. *americanus*, Gosselin *et al* [18] proposed that female choice and/or male competition could have been altered by effects of fisheries-induced sex ratio imbalance, which may have included sperm limitation. However, male and female abundance and size distributions are approximately equal in *H*. *gammarus* around Cornwall [42,55], which may serve to maintain the ubiquity of monandrous mating. Male density affects the frequency of multiple paternity in many species (e.g. house mice [56]; European earwig [57]), and if the proportion of breeding males were driving variation in the occurrence of multiple paternity in lobsters, the frequency of multiply-sired clutches could follow a Gaussian distribution; both even sex ratios and extreme male depletion would be expected to lead to single paternity, with multiple paternity most frequent in an intermediate state of partial male depletion. For example, male density explains a normally-distributed dynamic in the fertilisation success of female Red sea urchins [58]. Even if female lobsters were inseminated by multiple males, spermatophore stratification may ensure last-male precedence upon fertilisation, as is the case in Snow crabs [8].

Potential mechanisms preserving single paternity in Cornwall may be weakened or absent in other *H*. *gammarus* stocks, however. Further assessments of paternity would be particularly valuable in stocks recovering from collapse (e.g. Norway [24,43,59]), of limited size distribution (e.g. NE England [55]), of high abundance (e.g. Lundy, UK [60–62]) and in the absence of fishing (e.g. Lundy, UK; Flødevigen, Bolærne and Kvernskjær in Scandinavia [63]). If destabilised population demography were found to affect the frequency of multiple paternity, such data could be a useful reference point as to the health of lobster fisheries. Although *Homarus* species are presumed to be polygynous [21], we found no evidence of any male fertilising multiple clutches, despite some females within individual sample sites being captured in close proximity (i.e. traps approximately 100 m apart). Sex-biased conservation measures may result in sperm limitation [28], so knowledge on paternity and the fertilisation success of individual males would benefit fishery managers in ensuring conservation legislation safeguards recruitment.

The results of PrDM simulations suggest that a different sampling regime to that which we employed would enhance power to detect multiple paternity at highly uneven skews. Genotyping 10 eggs per clutch at 13 loci amplified in four multiplexes (40 PCR reactions) gave us an estimated 65% power to detect additional males contributing just 10% of fertilisations. However, PrDM was only slightly reduced by using only the three most informative loci, which can be multiplexed together. As such, the attainment of >95% power to detect secondary males in a 90:10 fertilisation skew would have been possible with a progeny array of 34 eggs per clutch, each genotyped in a single PCR reaction (34 PCR reactions). Although this would require more DNA extractions, it may be a preferable option in future studies of parentage using these microsatellites, assuming those loci are similarly diverse elsewhere. Especially where population allele frequencies are readily available, *a priori* analysis of PrDM would be advisable to determine the most efficient sampling regime and marker panel. Further attempts to genotype *H*. *gammarus* eggs would also be advised to avoid clutches in early phases of development to ensure only fertilised eggs are sampled and that DNA yields are sufficient for downstream analysis.

Our findings of high allelic diversity and single paternal fertilisations in this population of *H*. *gammarus* bodes well for the potential utility of genetic markers in parentage assignments [64] to enable evaluations of fisheries conservation measures, and particularly hatchery stocking. As a result of the recent collapses seen in some stocks and the increased fishing pressures on others, attempts have been made in a variety of European locations, including Cornwall [33], to enhance the productivity and sustainability of *H*. *gammarus* fisheries via the release of cultured juveniles [36,43,59,65,66]. Genetic tagging, the establishment of hatchery origin via multi-locus assignment of parentage, has important advantages over existing tagging options for juvenile lobsters, such as sub-lethal sampling and no restrictions on the body size of released individuals, as well as providing data for the assessment of genetic impacts on the wild target stock [36]. Hatcheries sourcing ovigerous lobsters from the wild may genotype maternal tissues directly, but paternal genotype(s) must be deduced from a sample of eggs or larvae in order to establish all possible progeny genotypes [36]. Since single paternity appears to be the regular mode of fertilisation in this region, the resolution of parentage may be achieved by genotyping many fewer progeny than would be required were multiple paternity frequent. As a result, the compilation of the anticipated genotypes of released lobsters, a necessary step before surveying the wild population, would be more affordable. The development of a genetic tagging approach may become a crucial tool with which to assess and compare different *H*. *gammarus* conservation strategies, particularly in light of the scarcity of methods with which to monitor recruitment and the performance of wild larvae and juveniles [21,36,67].

## Conclusions

Multi-locus genotyping proved a powerful tool in the assessment of paternity in *H*. *gammarus*, and provided evidence only of singly-sired clutches in an important regional population. Multiple paternity was not detected, indicating it is likely to be either absent, or irregular and highly skewed in favour of a single male. The detection of only single paternity among *H*. *gammarus* may reflect demographic stability in sex-ratios across a wide size distribution in this region. The development of additional microsatellite markers provides greater power for further studies of parentage and population genetics in *H*. *gammarus*. The prospects of their potential utility in evaluations of hatchery stocking and other fishery conservation measures in Cornwall are increased by the establishment of single paternity as the dominant method of fertilisation.

## Supporting Information

S1 Table Primer sequences of tested loci. Table featuring primer sequences of novel loci tested and cause of discard where development was not achieved.
(DOCX)

S2 Table Dataset of population genetic survey of novel microsatellite loci. Spreadsheet featuring scored genotypes of tissues collected from around the coast of Cornwall, UK. For each of 24 individuals sampled from 13 locations (n = 312), allele scores (in base pairs) are shown for the three microsatellite loci (HGD110, HGD117 and HGD129) developed during this investigation.
(XLSX)

S3 Table Characteristics of novel microsatellite loci. Three novel microsatellite loci with associated diversity information: *N*_A_ = number of alleles; *H*_E_ = expected heterozygosity; *H*_O_ = observed heterozygosity; H-W = p-values for deviation from Hardy-Weinberg equilibrium as evidenced by exact test (*p*) and U-test of heterozygote excess (*H*_ex_).
(DOCX)

S4 Table Dataset of maternal and progeny genotypes forming the paternity assays. Spreadsheet featuring scored genotypes of maternal and progeny tissues, and resolved paternal genotypes. For each of 34 assays of *H*. *gammarus* paternity, allele scores (in base pairs) are shown at 15 microsatellite loci (including HGA8 and HGC129, both0020later dropped for evidence of null alleles). Each assay features twelve samples; maternal genotype at top, followed by the genotypes of 10 progeny, with resolved paternal genotype at bottom (italicised). Sample names compose a letter denoting capture location and size (mm) of female lobster, followed by sample type, where M = maternal, P = paternal, and for progeny, 1–5 denotes pleopod region (with 5 nearest the tail), and t = tip or b = base of pleopod.
(XLSX)

S5 Table Estimates of PrDM at various paternity scenarios. Table shows calculations of the probability of detecting multiple paternal contributions (PrDM) and the number of egg genotypes required to achieve a 95% confidence level in PrDM. Values reflect various scenarios of numbers of sires and their fertilisation skew, and are calculated for all 13 loci (as used in this study) and the three most polymorphic loci (all from Multiplex 4). Predictions used allele frequencies obtained from a survey of 312 individuals in the south-western United Kingdom.
(DOCX)

S1 Text Microsatellite development. Methodology and results of the characterisation of novel microsatellite loci.
(DOCX)
