# Supplementary material for: Genotype Reconstruction of Paternity in European Lobsters (Homarus gammarus)
Source: PLoS One. 2015 Nov 13;10(11):e0139585. doi: 10.1371/journal.pone.0139585 (PMC4643931; doi:10.1371/journal.pone.0139585)
Supplement: S1 Table — Table featuring primer sequences of novel loci tested and cause of discard where development was not achieved. (DOCX) [file pone.0139585.s001.docx]

**S1 Table. Primer sequences of tested loci.** Table featuring primer sequences of novel loci tested and cause of discard where development was not achieved.

| **Locus** | **Primer** | **Primer sequence (5'-3')** | **Developed / Reason Undeveloped** |
| --- | --- | --- | --- |
| HGD110 | HGD110F | ACGGATGGATGGATAGGTAG | Developed |
|  | HGD110R | ATTCTCTGGCAGGTCAAGAC |  |
| HGD117 | HGD117F | GCCTACTCTCTCCTTCCTTC | Developed |
|  | HGD117R | ACCTGTCTATCGTTCTGTTTG |  |
| HGD129 | HGD129F | CCGTGCTGAAAGGGTTAT | Developed |
|  | HGD129R | CAAACTATTCGTCCACAAAGTC |  |
| HGA5 | HGA5F | GGTGTCCAGCAAACAATATAGG | Difficulty in consistent scoring |
|  | HGA5R | ACCTGCACTTGTACCCACAC |  |
| HGD121 | HGD121F | AGCAGATGTAACCGAGGTAGT | Difficulty in consistent scoring |
|  | HGD121R | GAATGAAGCACCATAACACAG |  |
| HGC107 | HGC107F | CTCTGCTCTTTCTGGTGTTG | Difficulty in consistent scoring |
|  | HGC107R | GTCGGCACTAAACTCATCAC |  |
| HGC121 | HGC121F | TCAACCTTTCCAGACAAGTGA | Appeared monomorphic |
|  | HGC121R | AGGAACGTAGACCCGTACAGAG |  |
| HGC106 | HGC106F | GATCGAACTCAGGTCCAC | Failed to amplify |
|  | HGC106R | TTTGTGTGTGTATGTGTG |  |
