# Supplementary material for: Genotype Reconstruction of Paternity in European Lobsters (Homarus gammarus)
Source: PLoS One. 2015 Nov 13;10(11):e0139585. doi: 10.1371/journal.pone.0139585 (PMC4643931; doi:10.1371/journal.pone.0139585)
Supplement: S3 Table — Three novel microsatellite loci with associated diversity information: N A = number of alleles; H E = expected heterozygosity; H O = observed heterozygosity; H-W = p-values for deviation from Hardy-Weinberg equilibrium as evidenced by exact test (p) and U-test of heterozygote excess (H ex). (DOCX) [file pone.0139585.s003.docx]

**S3 Table. Characteristics of novel microsatellite loci.** Three novel microsatellite loci with associated diversity information: *N*_A_ = number of alleles; *H*_E_ = expected heterozygosity; *H*_O_ = observed heterozygosity; H-W = p-values for deviation from Hardy-Weinberg equilibrium as evidenced by exact test (*p*) and U-test of heterozygote excess (*H*_ex_).

| **GenBank accession number** | **Locus** | **Primer sequence (5’-3’)** | **Repeat motif** | **Size range (bp)** | ***N*_A_** | ***H*_E_** | ***H*_O_** | **H-W**  ***p H*_ex_** | |
| --- | --- | --- | --- | --- | --- | --- | --- | --- | --- |
| KT240103 | HGD110 | F: ACGGATGGATGGATAGGTAG  R: ATTCTCTGGCAGGTCAAGAC | (AGAT)8 | 176-220 | 11 | 0.799 | 0.824 | 0.5637 | 0.201 |
| KT240104 | HGD117 | F: GCCTACTCTCTCCTTCCTTC  R: CCTGTCTATCGTTCTGTTTG | (ATAG)7 | 254-302 | 10 | 0.574 | 0.574 | 0.116 | 0.195 |
| KT240105 | HGD129 | F: CCGTGCTGAAAGGGTTAT  R: CAAACTATTCGTCCACAAAGTC | (AGAT)11 | 234-290 | 10 | 0.563 | 0.564 | 0.837 | 0.640 |
