# Supplementary material for: Genotype Reconstruction of Paternity in European Lobsters (Homarus gammarus)
Source: PLoS One. 2015 Nov 13;10(11):e0139585. doi: 10.1371/journal.pone.0139585 (PMC4643931; doi:10.1371/journal.pone.0139585)
Supplement: S5 Table — Table shows calculations of the probability of detecting multiple paternal contributions (PrDM) and the number of egg genotypes required to achieve a 95% confidence level in PrDM. Values reflect various scenarios of numbers of sires and their fertilisation skew, and are calculated for all 13 loci (as used in this study) and the three most polymorphic loci (all from Multiplex 4). Predictions used allele frequencies obtained from a survey of 312 individuals in the south-western United Kingdom. (DOCX) [file pone.0139585.s005.docx]

**S5 Table.** **Estimates of PrDM at various paternity scenarios.** Table shows calculations of the probability of detecting multiple paternal contributions (PrDM) and the number of egg genotypes required to achieve a 95% confidence level in PrDM. Values reflect various scenarios of numbers of sires and their fertilisation skew, and are calculated for all 13 loci (as used in this study) and the three most polymorphic loci (all from Multiplex 4). Predictions used allele frequencies obtained from a survey of 312 individuals in the south-western United Kingdom.

| **Paternal skew – two sires**  (Primary male : Secondary male) | | | | **50:50** | **60:40** | **70:30** | **80:20** | **90:10** |
| --- | --- | --- | --- | --- | --- | --- | --- | --- |
| **13 loci**  **(4 multiplexes)** | | **PrDM with 10 eggs** | | 0.998 | 0.993 | 0.970 | 0.891 | 0.649 |
|  |  | ***n* eggs for PrDM >0.95** | | 6 | 7 | 9 | 14 | 29 |
| **3 loci**  **(1 multiplex)** | | **PrDM with 10 eggs** | | 0.983 | 0.976 | 0.946 | 0.856 | 0.612 |
|  |  | ***n* eggs for PrDM >0.95** | | 8 | 8 | 11 | 17 | 34 |
| **Paternal skew – three sires**  (Primary male : Secondary males) | | | **34:33:33** | **50:25:25** | **60:20:20** | **70:15:15** | **80:10:10** | **90:5:5** |
| **13 loci**  **(4 mplxs)** | **PrDM with 10 eggs** | | 1.000 | 0.999 | 0.994 | 0.971 | 0.890 | 0.648 |
|  | ***n* eggs for PrDM >0.95** | | 5 | 5 | 6 | 9 | 14 | 29 |
| **3 loci**  **(1 mplx)** | **PrDM with 10 eggs** | | 0.998 | 0.996 | 0.986 | 0.955 | 0.862 | 0.616 |
|  | ***n* eggs for PrDM >0.95** | | 6 | 6 | 8 | 10 | 16 | 32 |
