# Supplementary material for: Genotype Reconstruction of Paternity in European Lobsters (Homarus gammarus)
Source: PLoS One. 2015 Nov 13;10(11):e0139585. doi: 10.1371/journal.pone.0139585 (PMC4643931; doi:10.1371/journal.pone.0139585)
Supplement: S1 Text — Methodology and results of the characterisation of novel microsatellite loci. (DOCX) [file pone.0139585.s006.docx]

**S1 Text. Microsatellite development.** Methodology and results of the characterisation of novel microsatellite loci.

To improve analytical power, novel loci were developed to complement the species-specific microsatellite panel already publicly available. To characterise new loci, eight tetra-repeat microsatellites, isolated from partial genomic libraries, were used to design primer pairs as described by André & Knutsen [1]. Preliminary marker tests were conducted by analysing 12 individuals (none included in paternity assays), four from each of three of the study sites; Tintagel, Sennen and Looe. Of these eight loci, five either failed to amplify (HGC106), appeared to be monomorphic (HGC121), or presented significant difficulties in scoring alleles consistently (HGA5, HGC107, and HGD121) (S1 Table). Further, comprehensive screening was conducted for the three loci that amplified reliably and were polymorphic (HGD110, HGD117 and HGD129). Comprehensive screening involved the analysis of 312 individuals; 24 from each of 13 geographic samples (including the four paternity sample sites; see Figure 1 in the main paper for locations) spanning 230 km of coastal waters from Looe (the south-eastern-most paternity sample site) to Boscastle (beyond the north-eastern-most paternity sample site) and west to the Isles of Scilly (offshore from the western-most paternity sample site). These samples were genotyped at the novel loci, as well as the existing 12 loci of André & Knutsen [1] to enable checks for linkage disequilibrium.

DNA extraction, PCR amplification and fragment analysis of loci followed the protocols listed in the Microsatellite Genotyping section in the main paper. Taq PCR Master Mix (Qiagen) used to amplify loci instead of Multiplex PCR Mix. Population differentiation among geographic samples was checked by G-tests in the web-based GENEPOP 4.2 software [2], to justify pooling samples as a single unit for the characterisation of novel loci, testing for null alleles, and the estimation of allele frequencies. Across all 15 loci, significant genic differentiation was detected among the 13 spatial samples, but not after the removal of HGA8 and HGC129, loci later found to be affected by null alleles. A G-test for overall population differentiation was then non-significant (p = 0.07), and only four of 91 sample pairs showed significant differentiation (p < 0.05), as expected by chance alone.

These genotypes were also tested in GENEPOP 4.2 for heterozygosity, linkage disequilibrium and deviation from Hardy-Weinberg expectations. All tests of linkage disequilibrium were non-significant after this threshold was adjusted to account for multiple tests [3]. No deviation from Hardy-Weinberg expectations were detected via the exact probability test (p = 0.30; [4]) or U-test of global heterozygote excess (p = 0.50; [5]). For the newly-developed loci HGD110, HGD117 and HGD129, genotyping of the 312 individuals from Cornwall (S2 Table) revealed that the number of alleles ranged from 10 to 11 and the observed heterozygosity was 0.56 to 0.82 (S3 Table). The likelihood of null alleles being present was estimated in the software FreeNA [6], which did not detect any failed amplification among alleles (estimated frequencies of null alleles were <0.0001 for all loci).

**References**

1. André C, Knutsen H. Development of twelve novel microsatellite loci in the European lobster (*Homarus gammarus*). Conserv Genet Resour*.* 2010; 2: 233-236.
2. Raymond M, Rousset F. GENEPOP (version 1.2): population genetics software for exact tests and ecumenicism. J Heredity. 1995; 86 (3): 248-249.
3. Benjamini Y, Hochberg Y. Controlling the false discovery rate: a practical and powerful approach to multiple testing. J Roy Stat Soc B Met. 1995; 57: 289-300.
4. Haldane JBS. An exact test for randomness of mating*.* J Genet. 1954; 52 (3): 631-635.
5. Rousset F, Raymond M. Testing heterozygote excess and deficiency. Genetics. 1995; 140 (4): 1413-1419.
6. Chapuis MP, Estoup A. Microsatellite null alleles and estimation of population differentiation. Mol Biol Evol. 2007; 24 (3): 621-631.
